# SnRK2s: Kinases or Substrates?

**DOI:** 10.3390/plants14081171

**Published:** 2025-04-09

**Authors:** Yunmin Wei, Linzhu Peng, Xiangui Zhou

**Affiliations:** 1 Key Laboratory of Plant Genetics and Molecular Breeding, Zhoukou Normal University, Zhoukou 466001, China; 2 College of Life Sciences and Oceanography, Shenzhen University, Shenzhen 518000, China; 2021300070@email.szu.edu.cn

**Keywords:** phosphorylation, post-translational modification, substrates, SnRK2

## Abstract

Throughout their life cycle, plants persistent through environmental adversities that activate sophisticated stress-signaling networks, with protein kinases serving as pivotal regulators of these responses. The sucrose non-fermenting-1-related protein kinase 2 (SnRK2), a plant-specific serine/threonine kinase, orchestrates stress adaptation by phosphorylating downstream targets to modulate gene expression and physiological adjustments. While SnRK2 substrates have been extensively identified, the existing literature lacks a systematic classification of these components and their functional implications. This review synthesizes recent advances in characterizing SnRK2-phosphorylated substrates in *Arabidopsis thaliana*, providing a mechanistic framework for their roles in stress signaling and developmental regulation. Furthermore, we explore the understudied paradigm of SnRK2 undergoing multilayered post-translational modifications (PTMs), including phosphorylation, ubiquitination, SUMOylation, S-nitrosylation, sulfation (S-sulfination and tyrosine sulfation), and N-glycosylation. These PTMs collectively fine-tune SnRK2 stability, activity, and subcellular dynamics, revealing an intricate feedback system that balances kinase activation and attenuation. By integrating substrate networks with regulatory modifications, this work highlights SnRK2’s dual role as both a phosphorylation executor and a PTM-regulated scaffold, offering new perspectives for engineering stress-resilient crops through targeted manipulation of SnRK2 signaling modules.

## 1. Introduction

Drought, high salinity, osmotic stress, cold, heat, and heavy metals are the primary environmental factors limiting plant growth and agricultural productivity. To adapt to these adverse conditions, plants have evolved a series of physiological and molecular mechanisms over evolutionary timescales. Among these, protein phosphorylation and dephosphorylation play pivotal roles in signal transduction during plant responses to abiotic stressors [1]. Protein phosphorylation is a reversible process mediated by two antagonistic enzymes: protein kinases and phosphatases. Protein kinases perceive diverse environmental signals and activate distinct phosphorylation cascades to regulate the downstream expression of the target gene, thereby enhancing plant resilience against various abiotic stresses. Key plant protein kinase families include receptor-like protein kinases (RLKs), mitogen-activated protein kinases (MAPKs), calcium-dependent protein kinases (CDPKs), and sucrose non-fermenting-1-related protein kinases (SnRKs).

*Arabidopsis thaliana* contains ten SnRK2 kinases, which are classified into three subfamilies: subfamily I (SnRK2.1, SnRK2.4, SnRK2.5, SnRK2.9, SnRK2.10), subfamily II (SnRK2.7, SnRK2.8), and subfamily III (SnRK2.2, SnRK2.3, OST1/SnRK2.6) [2,3,4,5,6]. Kinases in subfamily I exhibit no sensitivity to abscisic acid (ABA), kinases in subfamily II show weak or no induction by ABA, whereas kinases in subfamily III are strongly induced by ABA. Notably, SnRK2.2, SnRK2.3, and SnRK2.6 are key components of ABA signaling pathways [7,8,9]. In the absence of ABA, type-A protein phosphatases PP2Cs (e.g., ABI1) inhibit the kinase activity of SnRK2.2/2.3/2.6 [10,11,12,13]. Upon stress exposure, ABA accumulates and binds to PYRAB-ACTIN RESISTANCE1 (PYR1)/PYR1-LIKE (PYL)/REGULATORY COMPONENT OF ABA RECEPTOR (RCAR) receptors, forming a complex that interacts with PP2Cs to relieve their inhibitory effects on SnRK2.2/2.3/2.6. Activated SnRK2 kinases subsequently phosphorylate downstream substrates, positively regulating ABA-responsive pathways [11,12,13,14]. These three kinases exhibit functional redundancy; for example, the *snrk2.2/2.3/2.6* triple mutant displays stronger ABA insensitivity compared to single or double mutants [2,6,15]. While *snrk2.2/2.3* mutants exhibit strong resistance to ABA during seed germination and root growth, they show no significant difference in stomatal movement compared to wild-type plants. Conversely, the *snrk2.6* single mutant demonstrates ABA insensitivity in stomatal regulation, accompanied by increased water loss from detached leaves and heightened drought sensitivity. In the *snrk2.2/2.3/2.6* triple mutant, nearly all ABA-responsive downstream genes fail to be activated, resulting in severe ABA insensitivity during seed dormancy, germination, root growth, and stomatal movement. These findings underscore the central regulatory role of subfamily III SnRK2 kinases in ABA signaling [2,10].

Despite extensive studies on ABA-induced activation mechanisms of SnRK2.2/2.3/2.6, the functions and substrates of ABA-independent SnRK2s remain relatively underexplored. This gap is partly due to the high degree of functional redundancy within the SnRK2 kinase family. Single mutants often exhibit minimal developmental phenotypes, complicating the efforts to elucidate the physiological roles of individual members. Fujii et al. generated an *Arabidopsis* decuple mutant (*snrk2.1/2.3/3.4/4.5/5.6/6.7/7.8/8.9/9.10*) and demonstrated that SnRK2s are the primary protein kinases responding to osmotic stress in plants [16]. However, the precise mechanisms underlying the activation of these kinases by osmotic stress remain unclear [11], including potential interactions with known or unknown osmotic stress receptors/sensors. Addressing these questions represents a critical direction for future research.

In this review, we systematically summarize the target proteins of SnRK2s and classify them based on their biological functions, including ABA/stomatal movement, drought, osmotic stress, and cold stress. Additionally, we synthesize existing reports on post-translational modifications of SnRK2s mediated by other proteins.

## 2. SnRK2 and ABA

The phosphorylation and dephosphorylation of proteins serve as the switches for ABA responses, and the SnRK2 represents a key core component in ABA signal transduction. As a protein kinase, SnRK2 mainly classifies the target proteins it regulates in ABA signaling into two categories: transcription factors and ion channels. Research has demonstrated that downstream transcription factors such as ABF1 [9], ABF2/AREB1 [9,17,18], ABF3 [19], ABF4/AREB2 [17], and ABI5 [9,15] can all be directly phosphorylated by SnRK2, and they are among the earliest and, undoubtedly, one of the most classic and important components of ABA signaling (Table 1).

Numerous studies have demonstrated that the transcription factor RAV1 is a member of the RAV (related to ABI3/VP1) subfamily within the AP2/ERF (APETALA2/Ethylene responsive factor) transcription factor superfamily. RAV1 directly binds to the promoters of *ABI3*, *ABI4*, and *ABI5* to downregulate their expression. The SnRK2.2/2.3/2.6 kinases interact with and phosphorylate RAV1, thereby reducing RAV1’s inhibitory effect on *ABI5* [20].

ABA-responsive kinase substrate 1 (AKS1) is a basic helix-loop-helix (bHLH) transcription factor that typically forms multimeric complexes with DNA. Upon phosphorylation and exposure to ABA, AKS1 undergoes monomerization, which consequently diminishes its transcriptional activation activity [21]. Subsequent studies have indicated that AKS1, as an endogenous phosphorylation substrate, would be phosphorylated by SnRK2s in *Arabidopsis* guard cells in response to ABA [22]. SnRK2.6 can interact with RAP2.6 and phosphorylating it. RAP2.6 can directly bind to the GCC or DREs of *RD29A* and *COR15A*, thereby facilitating their expression [23].

Recent research has shown that SnRK2s play a role in stomatal precursor development and directly phosphorylate SPCH (the main transcription factor for stomatal initiation). SnRK2.2/2.3/2.6 kinases act on specific amino acid residues of SPCH, which mediate the inhibition of SPCH by ABA/drought and the suppression of stomatal formation [24].

In the ion channel aspect, the slow anion channel protein SLAC1 [25,26,27,28] and the quick anion channel protein QUAC1 [29] that are closely related to stomatal movement, as well as the potassium ion channel KAT1 [30] and chloride channels AtCLCa [31], are all regulated by SnRK2.6. Additionally, SnRK2.2 phosphorylates AHA2, a PLASMA MEMBRANE PROTON ATPASE [32].

In the recent years, new targets downstream of SnRK2 have been discovered. SnRK2.6 phosphorylates PIP2;1, regulating the stomatal response to ABA [33]. SnRK2.6 can interact with an ATPase in chromatin remodeling complex BRM (BRAHMA) and phosphorylate it in vivo, thereby activating the downstream transcription factor ABI5 [34]. Interestingly, BRM can also interact with the negative regulator of ABA-signaling PP2C, and further studies show that the phosphorylation of BRM by SnRK2.6 is inhibited by PP2C [34]. Additionally, Group C Raf-like protein kinase Raf36/Raf22 can also be phosphorylated by SnRK2.6, serving as a brake for ABA signaling in the downstream of SnRK2 [35].

The ABA-activated calcium channels in the guard cell plasma membrane of *Arabidopsis thaliana* are primarily composed of four members of the CNGC family: CNGC5, CNGC6, CNGC9, and CNGC12 (collectively referred to as CNGC5/6/9/12). ABA activates these CNGC proteins to generate cytosolic calcium signals, thereby inducing stomatal closure [36]. SnRK2.6 directly interacts with CNGC5/6/9/12, targeting their N-terminal regions. Researchers identified a conserved serine residue at the N-terminus of CNGC proteins as the phosphorylation site using proteomics and in vitro protein phosphorylation techniques. Further studies, using point mutations combined with electrophysiology and intracellular Ca^2+^ imaging, demonstrated that an S-to-A mutation at this conserved serine residue significantly inhibits the Ca^2+^ channel activity of CNGC5/6/9/12, while an S-to-D mutation significantly enhances it. Subsequent in vivo experiments revealed that in response to drought and ABA stimulation, plants activate the Ca^2+^ channel activity of CNGCs through SnRK2.6-mediated phosphorylation of the conserved N-terminal serine residue, leading to extracellular Ca^2+^ influx and regulation of cytosolic Ca^2+^ oscillations in guard cells, ultimately controlling stomatal movement. This study elucidates a Ca^2+^-dependent ABA signaling pathway and demonstrates that this pathway is coupled with another non-Ca^2+^-dependent signaling branch via SnRK2.6, forming an integrated ABA signal network [37].

The Zhu Jiankang team employed quantitative phosphorylated proteomics to identify multiple targets of SnRK2.6. The researchers compared the overall changes in phosphorylated peptides in WT and *snrk2.2/2.3/2.6* triple mutant seedlings after ABA treatment. ABA increased the phosphorylation of 166 peptides in WT seedlings and decreased the phosphorylation of 117 peptides. In the *snrk2.2/2.3/2.6* triple mutant, 84 of the 166 peptides (representing 58 proteins) failed to be phosphorylated or had a reduced degree of phosphorylation in ABA treatment. In vitro kinase experiments indicated that these 58 proteins could act as substrates for SnRK2s. SnRK2 substrates encompass proteins involved in flower timing regulation, RNA and DNA binding, miRNA and epigenetic regulation, signal transduction, chloroplast function, and numerous other cellular processes. Among them, seven proteins (enhancing the rich level of late embryogenesis abundant (EEL), small conductance mechanosensitive channel 9 (MSL9), superoxide dismutase 2 (FSD2), AREB3, flowering basic helix-loop-helix transcription factor 3 (FBH3), binding to tomato mosaic virus RNA 1 long chain (BTR1L), and chloroplast outer membrane translocon complex 159 (TOC159)) might be phosphorylated by reconstructed SnRK2.6, while another two (arginine/serine-rich Splicing factor 41 (RSP41) and histone deacetylase 2B (HD2B)) could not. These results imply that most of the 58 SnRK2.2/2.3/2.6-dependent phosphorylated proteins are direct substrates of SnRK2s [38]. Moreover, the team reported that SnRK2.6 can phosphorylate thousands of peptide segments in ABA signaling [39]. Similarly, Kazuo Shinozaki’s research group identified multiple substrates, including MPK1, SnRK2-substrate 1 (SNS1) [40]. This suggests that phosphoproteomic analysis can identify multiple potential substrates of SnRK2; however, the biological significance of these phosphorylated substrates requires further experimental validation.

**Table 1:** The substrate proteins that are phosphorylated and regulated by SnRK2 in the ABA signaling pathway.

Substrates	Locus	Phosphorylation Sites	Kinases	Description	Reference
ABF1	AT1G49720	Not determined	SnRK2.2/2.3	bZIP transcription factor	[8]
ABF2/AREB1	AT1G45249	Ser26, Ser86, Ser94	SnRK2.2/2.3/2.6/2.7/2.8	bZIP transcription factor	[8,17,18]
ABF3	AT4G34000	Thr145	SnRK2.6	bZIP transcription factor	[19]
ABF4/AREB2	AT3G19290	Ser39	SnRK2.2/2.3/2.6	bZIP transcription factor	[17]
ABI5	AT2G36270	Not determined	SnRK2.2/2.3/2.6	bZIP transcription factor	[9,15]
RAV1	AT1G13260	Not determined	SnRK2.2/2.3/2.6	AP2/B3 domain transcription factor	[20]
AKS1	AT1G51140	Ser284, Ser288, Ser290	SnRK2.2/2.3/2.7	bHLH transcription factor	[22]
RAP2.6	AT1G43160	Not determined	SnRK2.6	ERF/AP2 transcription factor	[23]
SPCH	AT5G53210	Ser240, Ser271	SnRK2.2/2.3/2.6	bHLH transcription factor	[24]
SLAC1	AT1G12480	Ser59, Ser86, Ser113, Ser120	SnRK2.6	Anion channel	[25,26,27,28]
QUAC1	AT4G17970	Not determined	SnRK2.6	Anion channel	[29]
KAT1	AT1G04710	Thr306, Thr308	SnRK2.6	Potassium channel	[30]
AtCLCa	AT5G40890	Thr38	SnRK2.6	Chloride channel	[31]
AHA2	AT4G30190	Thr947	SnRK2.2	P-type ATPase	[32]
PIP2;1	AT3G53420	Ser121	SnRK2.6	Aquaporin	[33]
BRM	AT2G46020	Ser1760, Ser1762	SnRK2.6	Chromatin-Remodeling ATPase	[34]
Raf22	AT2G24360	Ser81	SnRK2.6	MAP kinase kinase kinase	[35]
Raf36	AT5G58950	Ser145	SnRK2.6	MAP kinase kinase kinase	[35]
CNGC5	AT5G57940	Ser20	SnRK2.6	Ca^2+^ channel	[37]
CNGC6	AT2G23980	Ser27	SnRK2.6	Ca^2+^ channel	[37]
CNGC9	AT4G30560	Ser26	SnRK2.6	Ca^2+^ channel	[37]
CNGC12	AT2G46450	Ser13	SnRK2.6	Ca^2+^ channel	[37]
EEL	AT2G41070	Not determined	SnRK2.6	Transcription factor	[38]
MSL9	AT5G19520	Not determined	SnRK2.6	Mechanosensitive ion channel	[38]
FSD2	AT5G51100	Not determined	SnRK2.6	Fe superoxide dismutase	[38]
AREB3	AT3G56850	Not determined	SnRK2.6	bZIP transcription factor	[38]
FBH3	AT1G51140	Not determined	SnRK2.6	bHLH transcription factor	[38]
BTR1L	AT5G04430	Not determined	SnRK2.6	Binding to tomv RNA 1L	[38]
TOC159	AT4G02510	Not determined	SnRK2.6	GTPase	[38]
MPK1	AT1G10210	Not determined	SnRK2.6	MAP kinase	[40]
SNS1	AT1G26470	Ser43	SnRK2.6	Chromatin modification-like protein	[40]

## 3. SnRK2 and Drought

Drought has a significant impact on plant growth and crop yields. The ABA signaling pathway serves as the central hub enabling plants to respond to drought stress. It enhances drought tolerance through a multifaceted approach, including rapid stomatal regulation, gene reprogramming, and morphological adaptation. Single mutants of SnRK2.6 and double mutants of SnRK2.2/2.3 are highly sensitive to drought stress, hence, SnRK2.2/2.3/2.6 exert positive regulation in drought stress response. Multiple substrates have been identified to participate in SnRK2-mediated drought stress response.

K^+^ uptake transporter 6 (KUP6) is positively regulated by drought stress. SnRK2.6 interacts with KUP6 and phosphorylates its C-terminal region, enhancing its function under drought conditions [41] (Table 2). NTL6, a plant-specific NAC (NAM/ATAF1/2/CUC2) transcription factor, undergoes proteolytic cleavage in response to biotic stress, which is mediated by abscisic acid. NTL6 directly interacts with SnRK2.8, which primarily phosphorylates Ser-142 of NTL6, thereby modulating its activity. The drought resistance mediated by NTL6 is dependent on the interaction with SnRK2.8 [42]. Additionally, OST1 regulates ABA-mediated stomatal closure through proteins other than ion channels. The ubiquitin ligase RAFP34 (ring zinc-finger protein34)/CHYR1 (CHY zinc-finger and ring protein1 [CHYR1]) is regulating the ABA-induced stomatal closure, active oxygen production, and drought resistance process. CHCY1 is mainly expressed in the vascular tissue and guard cells. SnRK2.2/2.3/2.6 interacts with CHYR1 and phosphorylates the Ser-178 of CHYR1 protein. The *chyr1* mutant seed germination and stomatal closure are insensitive to ABA and have increased sensitivity to drought. Overexpression of CHYR1T178A or CHYR1T178D show drought-sensitive and drought-resistant phenotypes, respectively. This indicates that OST1 can regulate the ubiquitin ligase activity of CHYR1 through phosphorylation, thereby positively regulating the role of ABA signaling in stomatal closure [43].

In addition to the ABF family of transcription factors, researchers have identified AtHAT1, a HD-ZIP class transcription factor in *Arabidopsis*, as a substrate of SnRK2.3. HAT1 functions as a negative regulator of ABA biosynthesis and drought stress responses in *Arabidopsis*. Overexpression of AtHAT1 suppresses ABA synthesis and consequently diminishes plant drought tolerance. Phosphorylation of HAT1 by SnRK2.3 reduces its activity, thereby alleviating its negative regulatory effects on ABA signaling and drought response pathways [44].

ABA and cytokinin exert antagonistic effects in numerous developmental processes and environmental stress responses of plants. SnRK2.2, SnRK2.3, and SnRK2.6 directly interact with type A response regulator 5 (ARR5), a negative regulator of the cytokinin signaling pathway, and phosphorylate it. The phosphorylation of serine residues on the ARR5 protein by the SnRK2s enhances its stability. Consequently, plants with overexpression of ARR5 display ABA sensitivity and drought resistance. Additionally, B-type ARRs ARR1, ARR11, and ARR12 physically interact with the SnRK2s and inhibit the kinase activity of SnRK2.6. The *arr1/11/12* triple mutants are sensitive to ABA. Genetic analysis indicates that the SnRK2s are upstream of ARR5 and downstream of ARR1, ARR11, and ARR12, playing an important role in regulating ABA responses and drought resistance [45].

Researchers identified a drought-tolerant mutant, *ppd5-2*, from a T-DNA insertion mutant library of nuclear-encoded chloroplast protein in *Arabidopsis thaliana* through a screening method. Further studies disclosed that PPD5 interacts with and is phosphorylated by OST1. The phosphorylation of PPD5 by OST1 increases its protein stability, but does not influence its chloroplast localization [46].

SnRK2.6/OST1 mediates microtubule disassembly during ABA-induced stomatal closure in *Arabidopsis thaliana*. Researchers have identified MAP SPIRAL1 (SPR1) as a substrate of OST1. OST1 interacts with and phosphorylates SPR1 at Ser-6, promoting the dissociation of SPR1 from microtubules and driving microtubule disassembly. Compared to wild-type plants, *spr1* mutants exhibit significantly increased water loss and reduced ABA responses, including impaired stomatal closure and microtubule disassembly in guard cells. These phenotypes were restored by introducing the phosphorylated active form of SPR1. SPR1 positively regulates microtubule disassembly during ABA-induced stomatal closure, which is dependent on OST1-mediated phosphorylation. These results reveal a critical role for SPR1 in ABA signaling and highlight the specific interaction between ABA signaling components and microtubule-associated proteins (MAPs) [47].

RAF22 [(Rapidly Accelerated Fibrosarcoma)-like mitogen-activated protein kinase kinase kinase 22] physically interacts with ABI1 (ABA insensitive 1) and phosphorylates the Ser-416 residue of ABI1 to enhance its phosphatase activity. Additionally, ABI1 can also dephosphorylate to enhance the activity of RAF22, thereby inhibiting ABA signal transduction and maintaining plant growth under normal conditions. Under drought stress conditions, SnRK2.6/OST1 activated by ABA phosphorylates the Ser-81 residue of RAF22 to inhibit its kinase activity and thereby inhibit its enhancement of ABI1 activity [48].

SNF1-related protein kinase 2 (SnRK2) substrate 1 (SNS1) is a negative regulator of ABA and drought stress and can be phosphorylated by SnRK2 in vivo [40,49,50]. We have noticed that VARICOSE (VCS), an mRNA decapping activator, serves as a substrate for multiple peptide segments of SnRK2.2/2.3/2.6 [40]. Subsequently, two independent research groups have verified that VCS is a target protein of the SnRK2 GroupⅠfamily members [51,52]. Under drought stress conditions, multiple members of the SnRK2 Group I phosphorylate VCS, and the knockdown plants of VCS reduces its tolerance to drought stress [51]. Another research revealed that VCS and varicose-related (VCR) are both interactors and phosphorylation targets of SnRK2.5, SnRK2.6, and SnRK2.10. These three protein kinases phosphorylate VCS at Ser-645 and Ser-1156, and SnRK2.6 and SnRK2.10 also phosphorylate VCR at Ser-692 and Ser-680. The SnRK2 proteins of the GroupⅠfamily, VCS, and XRN4 are involved in the regulation of root growth under normal conditions and the modulation of root morphology under salt stress [52].

Non-ABA-activated *Arabidopsis* SnRK2s (SnRK2.10) not only regulate the plant’s response to salt stress but also modulate its sensitivity to dehydration. Through phosphoproteomic analysis, several potential SnRK2.10 substrates were identified, including dehydrins ERD10 and ERD14. In vitro experiments confirmed that SnRK2.10 phosphorylates ERD14 and Ser-79 of ERF14, altering its subcellular localization [53,54]. Additionally, non-ABA-dependent SnRK2.4 phosphorylates the water channel protein PIP2;1 at Ser-121 [55].

Furthermore, numerous proteins such as MLP43, SASP, and PIA1 can interact with one or more members of the SnRK2 family; however, there is a dearth of direct evidence regarding whether they serve as direct phosphorylation substrates of SnRK2s in the drought stress response [56,57,58].

**Table 2:** The substrate proteins that are phosphorylated and regulated by SnRK2 in the drought stress tolerance.

Substrates	Locus	Phosphorylation Sites	Kinases	Description	Reference
KUP6	AT1G70300	Thr-759	SnRK2.6	K^+^ uptake transporter	[41]
NTL6	AT3G49530	Thr-142	SnRK2.8	Transcription factor	[42]
CHYR1	AT5G22920	Thr-178	SnRK2.6	Ubiquitin E3 ligase	[43]
HAT1	AT4G17460	Not determined	SnRK2.3	Transcription factor	[44]
ARR5	AT3G48100	Ser-21, Ser-33, Ser-72, and Ser-117	SnRK2.2/2.3/2.6	Transcription repressor	[45]
PPD5	AT5G11450	Thr-283	SnRK2.6	PsbP-domain proteins	[46]
SPR1	AT2G03680	Ser-6	SnRK2.6	MAP SPIRAL1	[47]
Raf22	AT2G24360	Ser-81	SnRK2.6	MAP Kinase Kinase Kinase	[48]
SNS1	AT1G26470	Ser-43	SnRK2.6	Chromatin modification-like protein	[49]
VCS	AT3G13300	Not determined	SnRK2.4/2.10/2.1/2.5	VARICOSE	[51]
VCS	AT3G13300	Thr-644, Thr-645, Ser-1156	SnRK2.5	VARICOSE	[52]
VCS	AT3G13300	Thr-644, Thr-645, Ser-692, Ser-1156	SnRK2.6	VARICOSE	[52]
VCS	AT3G13300	Thr-645, Ser-692, Ser-1155, Ser-1156	SnRK2.10	VARICOSE	[52]
ERD10	AT1G20450	Ser-22/23/61/65/106/107/208, Thr-49/213/214/221	SnRK2.10	Dehydrin protein	[53]
ERD14	AT1G76180	Ser-21, Thr-26, Ser-78, Ser-79, Ser-136	SnRK2.10	Dehydrin protein	[53]
PIP2;1	AT3G53420	Ser-121	SnRK2.4/2.10/2.1/2.5	Aquaporin	[55]

## 4. SnRK2 and Cold

Apart from its significant role in drought and osmotic stress, the SnRK2 kinase also exerts a considerable influence in cold stress. The C-repeat (CRT)-binding factors (CBFs) or dehydration-responsive element (DRE)-binding protein (DREB) can bind to the cis-element of CRT/DRE and trigger the transcription of downstream COR genes, thereby enhancing cold tolerance [59,60,61]. ICE1 is the core transcription factor in cold stress and positively regulates the expression of CBFs. OST1 phosphorylates ICE1 at position Ser-278 and enhances the protein stability and transcription activation ability of ICE1 during cold stress, thereby strengthening plant cold tolerance [62,63] (Table 3). Meanwhile, MPK3/6 negatively regulates the protein stability of ICE1, thus reducing plant cold tolerance [64]. Subsequently, the research group reported that BTF3/BTF3L, PUB25/PUB26, ANN1, and PP2CG1 are also phosphorylation substrates of OST1 [65,66,67].

BTF3 and BTF3L (BTF3-like), the *β*-subunits of the nascent polypeptide-associated complex (NAC), are phosphorylated at position Ser-50 by OST1, and OST1 enhances the interaction between BTF3 and CBFs under cold stress [65]. Two U-box type E3 ubiquitin ligases, PUB25/26, plays a crucial role in cold stress responses. In the *pub25 pub26* double mutant, the levels of CBFs are significantly reduced compared to wild-type plants, leading to increased cold sensitivity. Further studies have shown that PUB25/26 interacts with MYB15 and participates in its degradation during the early stages of cold treatment. Additionally, the Thr-94 and Thr-95 sites of PUB25/26 serve as target sites of OST1. Cold-activated OST1 phosphorylates PUB25/26 at these sites, enhancing its E3 ligase activity and promoting the ubiquitination and degradation of MYB15. This process positively regulates the induction of CBFs under cold stress and contributes to plant cold tolerance [66].

Yang Shu-hua’s research group utilized biochemical, molecular genetics, and electrophysiological approaches to discover that the calcium ion transporter protein AtANN1 plays a crucial role in low-temperature-induced calcium signaling. Their findings indicate that in the atann1 knockout mutant, the low-temperature-induced [Ca^2+^]_cyt_ is significantly reduced compared to wild-type plants. Additionally, the expression levels of key cold-responsive transcription factors CBFs and their downstream target genes COR are decreased, leading to diminished cold tolerance in Arabidopsis. These results suggest that AtANN1 influences the influx of low-temperature-mediated calcium signals, thereby regulating the CBF-COR-dependent cold signaling pathway and positively modulating plant cold tolerance. Furthermore, low-temperature-activated OST1 phosphorylates Ser-289 of the AtANN1 protein, enhancing its calcium transport activity and calcium binding affinity, which in turn regulates the generation of low-temperature-induced calcium signals [67]. Additionally, the kinase activity of OST1 in cold stress can be inhibited by the phosphatases EGR2 and PP2CG1 [68,69]. EGR2, a phosphatase localized to the cell membrane, interacts with OST1 and weakens its kinase activity, negatively regulating cold stress [68]. Simultaneously, low temperature induces OST1 to phosphorylate Ser-365 of PP2CG1, resulting in a decrease in the protein phosphatase activity of PP2CG1 and significantly affecting plant cold tolerance [69].

Therefore, OST1 is a core protein kinase in the cold stress response, regulating its activity through phosphorylation to enhance protein stability and positively regulate the cold stress response.

## 5. SnRK2 and Other Vital Biological Processes

In addition to positively regulating responses to drought and cold stress, the SnRK2 kinase family is also extensively implicated in various other biological processes, such as salt stress tolerance, pathogen defense, reactive oxygen species (ROS) generation, cell wall biosynthesis, and microRNAs biogenesis. The following sections will address each of these functions in detail.

Plants have evolved sophisticated signaling mechanisms to redirect growth away from adverse environmental conditions that compromise yield. Root gravitropism, characterized by sodium ion gradient-dependent directional growth, plays a pivotal role in salt stress adaptation. Despite its early discovery, the molecular basis of gravitropic responses under saline conditions remained elusive. Recent studies demonstrate that abscisic acid (ABA)-mediated root curvature governs *Arabidopsis* gravitropism through SnRK2.6 kinase activation. ABA-activated SnRK2.6 phosphorylates SP2L at Ser-406 induce asymmetric cell expansion in the root transition zone via cortical microtubule reorganization. Salt stress initiates SP2L-dependent reorientation of cell wall microtubules, which template cellulose microfibril deposition patterns. These structural modifications drive anisotropic cell wall extension, determining root tip curvature orientation. This molecular cascade elucidates how microtubule-guided microfibril alignment mediates differential cellular expansion, establishing a mechanistic framework for root salt-avoidance behavior. Crucially, salt stress triggers ABA-dependent SnRK2.6 activation, which phosphorylates the microtubule-associated protein SP2L to regulate microtubule array dynamics. This post-translational modification ultimately controls cellulose synthesis directionality and cellular expansion polarity in the root transition zone, enabling directional root growth away from saline substrates [70] (Table 4).

The MYB transcription factor NIGT1.4 is an important component in maintaining root growth under salt stress. T-DNA knockout mutation and functional complementation experiments have verified that NIGT1.4 functions in promoting the growth of the main root under salt stress. NaCl treatment induces the expression of NIGT1.4 in roots in an ABA-dependent manner. SnRK2.2 and SnRK2.3 interact with NIGT1.4 and phosphorylate it. The main root growth phenotype of the *snrk2.2/2.3/2.6* triple plant is like that of the *nigt1.4* mutant which both demonstrating salt stress sensitivity [71].

In plants the site infected by pathogens will exhibit necrotic lesions, and subsequently induce systemic acquired resistance (SAR) in distant tissues. NPR1 is a key regulator of SAR induced by SA. SnRK2.8 phosphorylates NPR1 and is indispensable for NPR1 to enter the nucleus. In *Arabidopsis*, the development of systemic immunity is mediated by SA signaling and SnRK2.8-mediated phosphorylation, which synergistically activate NPR1 in a double-reaction manner [72]. Later, it was confirmed that SnRK2.8-mediated phosphorylation of NPR1 is also necessary for NPR1 to enter the nucleus at low temperatures [73]. Besides phosphorylating NPR1, SnRK2.8 can also phosphorylate the effector protein AvrPtoB. SnRK2.8 interacts with AvrPtoB in yeast and plants. SnRK2.8 is essential for the virulence functions of AvrPtoB, including promoting bacterial colonization, inhibiting pectin deposition, and targeting plant defense regulatory factors NPR1 and receptor FLS2 [74].

Previous studies have shown that SnRK2.6/OST1 directly interacts with RBOHD and RBOHF [75]. OST1 phosphorylates Ser13 and Ser174 of AtRBOHF, triggering the production of ROS in guard cells, leading to stomatal closure and enhanced plant tolerance to drought and salt stress [76]. The activity of AtRBOHF can also be regulated by phosphorylation by CIPK11 and CIPK26 [77]. Similarly, Ser-343 and Ser-347 of AtRBOHD are targets of OST1, which are crucial for plant cell-to-cell active oxygen signaling under high light stress [78,79].

NRT1.1/NPF6.3/chlorate-resistant 1 (CHL1) is the first identified nitrate transporter, and it is surprising that SnRK2.2/2.3/2.6 interacts with NRT1.1 in vitro and in vivo, and phosphorylates Ser-585 of NRT1.1. Phosphorylation of NRT1.1 by SnRK2s leads to a significant reduction in nitrate uptake and affects root growth [80].

In *Arabidopsis*, the formation of plant secondary cell wall (SCW) is reduced due to genetic blockage of ABA synthesis and perception. SnRK2.2/2.3/2.6 can interact with AtNST1 and phosphorylate it, where AtNST1 is a master regulatory factor that enhances SCW formation and lignin deposition in the stem fiber region. SnRK2-mediated phosphorylation mutations at the regulatory sites of AtNST1 would eliminate the regulatory function of this transcription factor [81].

MicroRNA (miRNA), a 20–24 nucleotide non-coding RNA ubiquitous in eukaryotes, regulates mRNA splicing/translation and governs plant developmental processes and stress responses. The miRNA biogenesis machinery relies on core components including DCL1 (type III ribonuclease), SE (zinc finger protein), and HYL1 (dsRNA-binding protein). While phytohormone ABA and osmotic stress signaling are known to modulate miRNA accumulation, their mechanistic interplay remained unclear. The Zhu Jiankang research group revealed that SnRK2 kinases—central regulators of ABA and osmotic stress pathways—directly orchestrate miRNA synthesis by phosphorylating core biogenesis components. *snrk2.2/2.3/2.6* mutants exhibited reduced miR160 and related miRNAs, accompanied by elevated pri-miRNA levels and target gene expression. Notably, HYL1 protein abundance declined in snrk2 triple/decuple mutants. Phosphorylation assays further implicated HYL1 and SE as SnRK2 substrates. This work elucidates SnRK2-mediated phosphorylation as a molecular bridge linking ABA/osmotic stress to miRNA biogenesis regulation [82].

**Table 4:** The substrate proteins that are phosphorylated and regulated by SnRK2 in the stress network.

Substrates	Locus	Phosphorylation Sites	Kinases	Description	Reference
SP2L	AT1G50890	Ser-406	SnRK2.6	Microtubule associated protein	[70]
NIGT1.4	AT1G13300	Not determined	SnRK2.2/2.3	MYB transcription factor	[71]
NPR1	AT1G64280	Ser-589, Thr-373	SnRK2.8	NONEXPRESSER OF PR GENES 1	[72]
AvrPtoB		Ser-258	SnRK2.8	*Pseudomonas* effector	[74]
RBOHD	AT5G47910	Ser-163	SnRK2.6	NADPH oxidase	[76]
RBOHF	AT1G64060	Ser-13, Ser-174	SnRK2.6	NADPH oxidase	[76]
RBOHD	AT5G47910	Ser-343, Ser-347	SnRK2.6	NADPH oxidase	[78]
NRT1.1	AT1G12110	Ser-585	SnRK2.2/2.3/2.6	Nitrate transporter	[80]
NST1	AT2G46770	Ser-316	SnRK2.2/2.3/2.6	NAC transcription factor	[81]
HYL1	AT1G09700		SnRK2.4/2.6	Hyponastic leaves 1	[82]
SE	AT2G27100		SnRK2.4/2.6	Serrate	[82]

## 6. Post-Translational Modification of SnRK2

SnRK2s not only serves as a substrate for protein kinase phosphorylation, but it can also act as a target and present various post-translational modifications, including phosphorylation, SUMOylation, ubiquitination, S-nitrosylation, sulfation, and glycosylation.

### 6.1. Phosphorylation

In the same year, research groups led by Wang Pengcheng in China, Julian Schroeder in the United States, and Yamaguchi-Shinozaki in Japan published research papers in Nature Communications, revealing that the B subfamily of RAF protein kinases mediates the phosphorylation and activation of SnRK2 [83,84]. Wang Pengcheng’s research group discovered that some B2/3 subfamily RAF protein kinases phosphorylate and activate SnRK2.2/2.3/2.6, while the B4 subfamily RAF protein kinases phosphorylate and activate the other six ABA-independent SnRK2s [83]. The Yamaguchi-Shinozaki’s research group discovered that three B4 Raf-like MAPKKKs (RAF18, RAF20, and RAF24) in *Arabidopsis* can interact with Group I SnRK2 proteins and phosphorylate and activate the Group I SnRK2s that are not responsive to ABA in stress conditions, but these three B4 Raf-like MAPKKKs are not activated by ABA and do not participate in the activation of Group III SnRK2s [84]. Julian Schroeder’s research group discovered that three B3 Raf-like MAPKKKs (MAPKKK δ1, MAPKKK δ6, and MAPKKK δ7) can phosphorylate the Ser-171 residue of SnRK2.6, which cannot be self-phosphorylated to activate it, to reactivate SnRK2.6 [85]. These three studies jointly disclose the crucial role of the RAF-SnRK2 kinase cascade in osmotic stress and ABA signaling pathways. Other reported RAF kinases involved in the phosphorylation of SnRK2 encompass ARK1/2/3 and Raf10 [86,87]. The RAF family of kinases pertains to the MAP kinase kinase kinase, and RAF mediates the self-activation of SnRK2 through phosphorylation to commence the activation process of SnRK2. These studies connect the MAPK cascade and SnRK2 kinases, suggesting that the regulation among different kinases might be a universal mechanism within cells.

Furthermore, it has been reported that the protein kinases involved in the phosphorylation of the SnRK2 complex encompass BAK1, BIN2, ARK, HT1, and so on. BAK1 forms a complex with other lysine-rich repeat receptor-like kinases (LRR-RLKs), such as FLS2 and EFR1, which are implicated in plant basal immune responses triggered by flg22 and elf18 or elf26 [88,89]. Researchers have discovered that the BRI1-associated Receptor Kinase 1 (BAK1) mutant loses water more rapidly than the wild type and is insensitive to ABA-induced stomatal movement. ABA treatment fails to induce the expression of OST1 and the production of ROS in the *bak1* mutant. The overexpression of OST1 cannot complementation for the insensitivity of *bak1* to ABA. BAK1 forms a complex with OST1 and phosphorylates it, and ABA treatment leads to an increase in the BAK1/OST1 complex, thereby enhancing downstream signaling. This suggests that BAK1 mediates the ABA-induced stomatal movement process via OST1 [90].

The research group led by Gong Zhizhong identified that the *Arabidopsis* BRI1-associated Receptor Kinase 1 (BAK1) loss-of-function mutant, *bak1*, exhibits hypersensitivity to ABA in seed germination and primary root growth. Specifically, the ABA-induced OST1 activity in the *bak1-4* mutant is significantly higher than in the wild type, accompanied by elevated transcription levels of downstream ABA-responsive genes. These findings suggest that BAK1 negatively regulates core ABA signaling output. This conclusion contrasts with the earlier report, which indicated that BAK1 promotes OST1 activity [89]. Moreover, the *bak1* mutant displays distinct responses to ABA in seed germination and primary root growth compared to the wild type. Activated BAK1 can phosphorylate OST1 at the Thr-146 site, directly inhibiting its kinase activity. Additionally, BAK1 can phosphorylate ABI1 within the PYR1-ABA-ABI1 complex, releasing inhibited ABI1 and further negatively regulating core ABA signaling [91].

*Arabidopsis thaliana* Glycogen synthase kinase 3 (GSK3)-like kinases exert significant roles in plant growth and development, as well as in stress responses. BIN2 interacts with the members of Group III subfamily of SnRK2 kinases and phosphorylates SnRK2.2 at Thr-181 and Thr180 of SnRK2.3, and enhances the kinase activity of SnRK2.3, thereby exerting a positive regulatory effect in ABA signaling [92].

Casein kinase 2 (CK2) regulates the SnRK2 kinase by phosphorylating several conserved serines in the ABA box of the SnRK2 protein, enhancing its interaction with the negative regulatory factor PP2C in the core ABA signaling module [93].

HT1 (high leaf temperature 1) 13 and carbonic anhydrase (CA) 17 are two constituents that seemingly are specifically involved in the carbon dioxide sensing pathway. The *HT1* gene encodes a protein kinase primarily expressed in guard cells and functions as a major negative regulator of CO_2_-induced stomatal closure. Intriguingly, HT1, a negative regulator of CO_2_-induced stomatal closure, is capable of phosphorylating OST1 and inhibiting the phosphorylation of SLAC1 by OST1 [94].

SnRK2s also partake in the response to high Mg^2+^ concentration stress. CIPK26 (CBL-Interacting Protein Kinase 26) was identified through immunoprecipitation and liquid chromatography-tandem mass spectrometry analysis as a protein interacting with SnRK2.2. CIPK3, CIPK9, and CIPK23 also interact with SnRK2.2 in vivo. In vitro experiments demonstrated that CIPK26 can phosphorylate and activate SnRK2.2. Under high Mg^2+^ conditions, both the *snrk2.2/2.3/2.6* triple mutant and the *cipk26/3/9/23* quadruple mutant present similar phenotypes of shorter aboveground parts [95].

Terrestrial plants employ evolutionarily conserved osmostress adaptation mechanisms, including the activation of SnRK2s, ABA accumulation, and ABA-dependent signaling. As osmotic stress responses often antagonize growth, these pathways are suppressed under non-stress conditions through clade A protein phosphatase 2Cs (PP2Cs), which act as negative regulators by constitutively binding to SnRK2s. PP2Cs maintain SnRK2s in an inactive state via dephosphorylation of conserved serine residues in their activation loops and steric obstruction of catalytic sites. During osmotic stress or ABA signaling, PP2C-mediated inhibition must be relieved to enable SnRK2 activation. The Zhao Yang group demonstrated that *Arabidopsis* receptor-like cytoplasmic kinase BIK1 orchestrates this regulatory switch by phosphorylating SnRK2.6. While the dominant *abi1-1* mutation (G180D) disrupts PYL-PP2C interactions and impairs PYL-mediated SnRK2 release, BIK1 bypasses this defect by directly phosphorylating SnRK2.6 at two critical tyrosine residues (Tyr-163/182). This phosphorylation event likely disrupts PP2C binding by interfering with the tryptophan “lock-and-key” interface between PP2Cs and SnRK2.6. Phenotypic analysis revealed that *bik1* mutants exhibit compromised SnRK2 activation, attenuated stress-responsive gene expression, reduced ABA biosynthesis, impaired growth homeostasis, and accelerated water loss under osmotic challenge [96].

Osmotic stress perception and signaling involve intricate mechanisms, with transient cytosolic Ca²⁺ elevation and rapid SnRK2 kinase activation representing primary early responses. Leveraging wild-type Arabidopsis (Col-0) and a *pyl* duodecuple mutant deficient in ABA receptors, Zhao Yang’s team employed quantitative phosphoproteomics to identify 19 putative early-responsive kinases under osmotic challenge. Among these, CPK3/4/6/11/27 emerged as Ca²⁺-dependent decoders of osmotic stress, exhibiting activation upon mannitol treatment or dehydration. Functional analyses revealed enhanced SnRK2 activation in CPK3/4/6/11/27 overexpression lines versus impaired responses in *cpk3/4/6/11/27* loss-of-function mutants. Mechanistically, CPKs phosphorylate seven conserved residues (S164/S166/S167/S171/S175/T176/T179) within SnRK2’s activation loop, with mutational studies confirming these phosphorylation events as essential for SnRK2.6 catalytic activity and physiological function. These findings establish CPK3/4/6/11/27 as critical mediators linking Ca²⁺ signaling to SnRK2 activation through multisite phosphorylation during osmotic stress adaptation [97].

### 6.2. Ubiquitination

A research team has discovered that SnRK2.3 can be ubiquitinated and degraded by AtPP2-B11, an F-BOX protein that is part of the SKP1/Cullin/F-BOX E3 ubiquitin ligase complex. The expression of *AtPP2-B1* is induced by ABA, and mutants with downregulated *AtPP2-B11* expression show hypersensitivity to ABA during seed germination and seedling growth. Overexpression of *AtPP2-B11* suppresses the ABA-hypersensitive phenotype of SnRK2.3 overexpression plants. This suggests that AtPP2-B11 can specifically degrade SnRK2.3 to negatively regulate plant responses to ABA [98]. Subsequently, researchers have also revealed that HOS15 (high osmotic stress 15) can facilitate the ubiquitination of SnRK2.6, leading to its degradation by the 26S proteasome. HOS15 is a substrate-receiving protein within the CUL4-DDB1 E3 ubiquitin ligase complex. The *hos15* mutants exhibit enhanced stability of the OST1 protein and are hypersensitive to ABA, showing strong tolerance to drought stress. The absence of OST1 function can significantly restrain the drought-sensitive phenotype of *hos15*. Simultaneously, ABA inhibits the interaction between HOS15 and OST1, thereby enhancing the stability of OST1. Moreover, ABI1 and ABI2 can promote the interaction between HOS15 and OST1 by dephosphorylating OST1 and subsequently facilitating its ubiquitination and degradation [99]. In later reports, there is direct evidence indicating that ubiquitin can interact directly with SnRK2.2/2.3 and inhibit their kinase activity [100].

### 6.3. SUMOylation

In 2022, two independent research groups almost concurrently reported that the SUMO protease ESD4 and its interacting protein NUA (nuclear pore anchor) regulate the stability of SnRK2.6/OST1 through deSUMOylation, thereby negatively modulating the ABA signaling pathway. In vitro SUMOylation experiments demonstrated that the SnRK2.6 protein can undergo SUMOylation, while ESD4 reduces the SUMOylation level of SnRK2.6. Hence, NUA and ESD4 might be capable of reducing protein stability by means of deSUMOylation [101,102]. Meanwhile, SnRK2.6/OST1 is also degraded by the HOS15 ubiquitin ligase via ubiquitination. Thus, it is hypothesized that SUMOylation is likely to enhance protein stability by competing with ubiquitin for binding to lysine residues on the protein.

### 6.4. S-Nitrosylation

ABA induces the generation of nitric oxide (NO) in guard cells, and the 137th cysteine residue near the kinase catalytic site of the OST1 protein can be sulfhydryl nitrosylated. The loss of function of the glutathione S-transferase omega (GSNOR) would give rise to the accumulation of nitric oxide in *gsnor1–3* mutant guard cells, leading to constitutive sulfhydryl nitrosylation of OST1 and ultimately blocking ABA-induced stomatal closure. Transforming the 137th cysteine residue of OST1 to serine and introducing it into the *gsnor1–2 ost1–3* double mutant can partially restrain the phenotype of *gsnor1–2* mutant guard cells that fail to close their stomata in response to ABA treatment. This indicates that nitric oxide can inhibit the kinase activity of OST1 via the sulfhydryl nitrosylation mechanism, thereby participating in ABA-mediated stomatal closure [103,104].

### 6.5. S-Persulfidation

Hydrogen sulfide (H_2_S), a water-soluble gas, plays a crucial role in regulating plant responses to environmental stress and growth. The formation of excessive sulfhydryl groups (with the conversion of Cys-SH to Cys-SSH), caused by the post-translational modification (PTM) of Cys residues and known as persulfidation, is directly regulated by H_2_S. Protein persulfidation proteomics data indicate that the SnRK2.6 protein undergoes persulfidation modification. Two persulfidation modification sites have been identified in SnRK2.6, and it has been discovered that these two Cys residues are exposed on the surface of SnRK2.6 and are adjacent to the catalytic ring and a key phosphorylation site of the kinase. Research reveals that when these two Cys residues are persulfidation-modified, they enhance the activity of SnRK2.6 and its interaction with downstream transcription factors of the ABA signal transduction. Furthermore, when Cys131, Cys137, or both were partially or completely replaced with serine in SnRK2.6C131S, SnRK2.6C137S, or SnRK2.6C131SC137S, these partially or fully substituted proteins were unable to restore the *ost1–3* mutant phenotype, displayed a reduced sensitivity to ABA and H_2_S-induced stomatal closure and Ca^2+^ influx, increased water loss, and decreased drought tolerance. The persulfidation reaction of SnRK2.6 has been demonstrated to positively regulate ABA signal transduction in guard cells. Therefore, this study proposed a novel mechanism for regulating the ABA signaling pathway, where H_2_S positively regulates ABA signal transduction in guard cells through persulfidation of SnRK2.6 [105]. In the subsequent year, the research group reported that hydrogen sulfide (H_2_S)-mediated S-persulfidation can modify the structure of the key kinase protein SnRK2.6 in the ABA signaling pathway, thereby enhancing its efficiency in transferring ATP-*γ*-phosphate groups and leading to increased kinase activity. The study also demonstrated that the phosphorylation level at critical sites of the SnRK2.6 protein positively regulates H_2_S-mediated S-sulfhydration [106]. The proposed mechanism of interaction between post-translational modifications not only provides novel insights into the field of protein post-translational modification but also offers theoretical support for understanding plant drought tolerance mechanisms [107,108].

### 6.6. Tyrosine Sulfation

In recent reports, a novel mechanism for ABA signal transduction “brake/desensitization” via tyrosine sulfation modification has been disclosed. Tyrosylprotein sulfotransferase (TPST) catalyzes the sulfhydryl (-SO_3_H) modification of the tyrosine residue (Y) in the substrate protein, thereby regulating the activity, stability, and protein–protein interactions of the substrate protein. There is only one TPST member in the *Arabidopsis* genome, and this study found that *Arabidopsis tpst* mutants are hypersensitive to ABA and that the ABA signal transduction pathway is overly activated in these mutants. Further studies demonstrated that TPST interacts with and sulfhydrylates the key kinase SnRK2.2/2.3/2.6 in the ABA signal transduction pathway, resulting in a significant reduction in the stability of the sulfhydrylated SnRK2.2/2.3/2.6 proteins and their rapid degradation through the 26S proteasome pathway, thereby reducing the intensity of ABA signal transduction and preventing ABA signals from being overly activated for an extended period of time [109].

### 6.7. N-Glycosylation

The most recent research outcomes indicate that SnRK2s can also undergo N-glycosylation modification. Through mutant screening, researchers found that mutant enzymes for N-glycosylation modification display a sensitive phenotype when exposed to exogenous ABA. They further discovered that the expression of N-glycosylation modification enzymes increased when plants were treated with ABA for an extended period. Subsequently, by employing molecular biology and cell biology techniques, they determined that prolonging the ABA treatment time enables N-glycosylation modification enzymes to bind to SnRK2.2/2.3 and carry out N-glycosylation modification on them. SnRK2.2/2.3 is a key factor in the ABA signal transduction pathway. When plants are treated with ABA for a short duration, SnRK2.2/2.3 mainly localizes in the nucleus of cells, activating the expression of ABA response genes and rapidly enhancing plant tolerance to stress. When the ABA treatment time is prolonged, SnRK2.2/2.3 undergoes N-glycosylation modification and gradually transitions from a nuclear localization to a peroxisome localization [110].

## 7. Conclusions and Future Prospects

**Figure 1:**
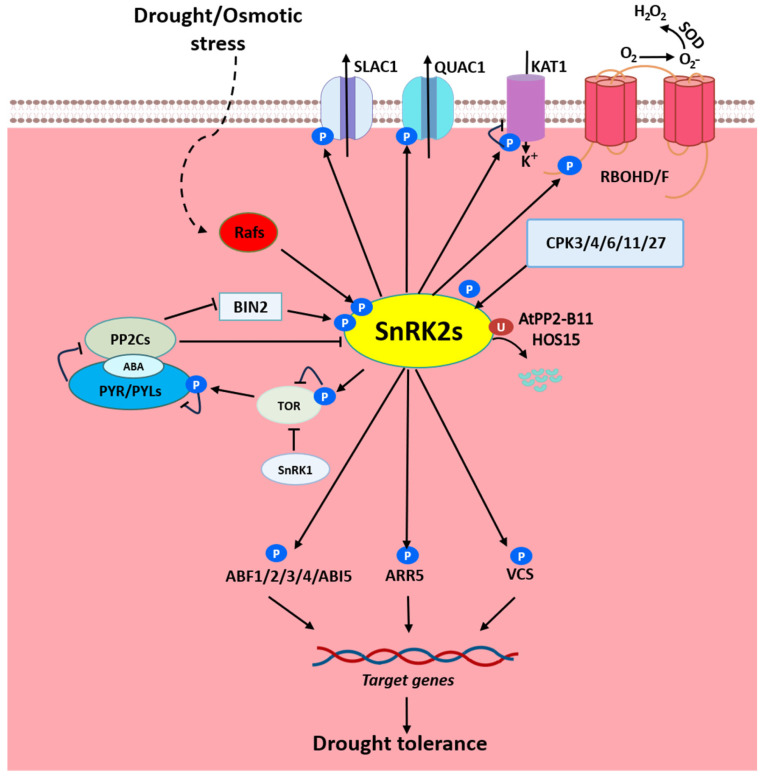
Mechanism of SnRK2 as the core protein kinase in ABA signaling during drought stress. Under ABA-absent conditions, the PP2C phosphatase inhibits the activity of the SnRK2 kinase via dephosphorylation, maintaining SnRK2 in an inactive state. Upon ABA binding, the affinity of PYL receptors for PP2C is enhanced, leading to the formation of the ABA-PYL-PP2C ternary complex. This complex directly suppresses the phosphatase activity of PP2C, thereby relieving its inhibition of SnRK2. Once freed from PP2C suppression, SnRK2 is activated through autophosphorylation or phosphorylation by other kinases (e.g., Raf, CPK, BIN2). The activated, phosphorylated SnRK2 translocates into the nucleus, where it phosphorylates the transcription factors (e.g., ABF1/2/3/4, ABI5, ARR5) and the P-BODY component VCS, driving the expression of stress-responsive genes to enhance plant drought tolerance. Simultaneously, SnRK2 phosphorylates ion channel proteins such as SLAC1, QUAC1, KAT1 (regulating stomatal closure) and RBOHD/F (modulating ROS production). Additionally, SnRK2 activity is regulated by post-translational modifications, including ubiquitination mediated by HOS15 and PP2-B11. This integrated mechanism coordinates gene expression, ion transport, and ROS signaling to bolster plant adaptation to drought stress.

The SnRK2 kinase family serves as a central regulatory node governing plant responses to abiotic and biotic stresses as well as developmental processes. This review comprehensively analyzes the known phosphorylation targets of SnRK2 kinases, establishing a functional framework wherein these kinases orchestrate diverse physiological outcomes through substrate-specific phosphorylation cascades (Figure 1). Notably, current research disproportionately focuses on ABA-responsive members (SnRK2.2/2.3/2.6), leaving seven ABA-independent isoforms largely uncharacterized. Given their established importance in osmotic stress adaptation, systematic investigation of these understudied kinases is imperative to elucidate their potential roles in alternative signaling networks.

Emerging evidence suggests SnRK2 kinases as master regulators of stress signaling, stomatal dynamics, redox homeostasis, and developmental plasticity. The ABA-SnRK2 axis exhibits extensive crosstalk with various phytohormone pathways including auxin, cytokinin, ethylene, and brassinosteroid signaling. Future studies should prioritize mapping these interactive networks to decode SnRK2’s multifaceted regulatory potential beyond canonical osmotic stress responses. While transcription factors dominate the current substrate inventories, critical gaps persist in characterizing SnRK2-mediated phosphorylation of major TF families (WRKY, ERF, MYB, NAC, bHLH). Systematic validation of candidate targets identified through phosphoproteomics, using orthogonal approaches like in vitro kinase assays and site-directed mutagenesis, will clarify their functional relevance in both ABA-dependent and independent contexts.

Intriguingly, SnRK2 proteins undergo complex post-translational modifications, revealing layered regulatory mechanisms that fine-tune kinase activity. SUMOylation enhances the stability of SnRK2 kinase, mitigates its degradation risk, and modulates its nucleocytoplasmic shuttling, thereby facilitating interactions between SnRK2 and nuclear targets such as transcription factors. Conversely, ubiquitination restricts excessive accumulation of SnRK2, preventing energy depletion or cellular damage caused by prolonged activation. Glycosylation further enables SnRK2 to integrate carbon metabolism with stress signaling, thus maintaining a dynamic equilibrium between growth and stress responses. The concerted action of multiple post-translational modifications establishes a “modification code”, equipping SnRK2 with the capacity to discern diverse signal inputs, including drought, salt stress, ABA signaling, and ROS. Leveraging this knowledge, gene editing technologies can be employed to target SnRK2 post-translational modification sites (e.g., enhancing SUMOylation for stability or inhibiting ubiquitination to reduce degradation), offering innovative strategies for developing drought-resistant and salt-tolerant crops.

In summary, the diverse post-translational modifications of SnRK2 kinases form an intricate regulatory network, enabling plants to exhibit rapid, precise, and reversible responses to changing environments. Future studies should focus on elucidating the spatiotemporal dynamics of these modifications, the mechanisms underlying their cross-talk, and their potential applications in enhancing stress resilience in crops.

## Figures and Tables

**Table 3:** The substrate proteins that are phosphorylated and regulated by SnRK2 in the cold stress tolerance.

Substrates	Locus	Phosphorylation Sites	Kinases	Description	Reference
ICE1	AT3G26744	Ser-278	SnRK2.6	Transcription factor	[62]
BTF3	AT1G17880	Not determined	SnRK2.6	NAC transcription factor	[65]
BTF3L	AT1G73230	Ser-50	SnRK2.6	NAC transcription factor	[65]
PUB25	AT3G19380	Thr-95	SnRK2.6	E3 ligase	[66]
PUB26	AT1G49780	Thr-94	SnRK2.6	E3 ligase	[66]
ANN1	AT1G35720	Ser-289	SnRK2.6	Calcium transporter	[67]
EGR2	AT5G27930	Not determined	SnRK2.6	E growth-regulating 2	[68]
PP2CG1	AT2G33700	Ser-365	SnRK2.6	Protein phosphatase 2C	[69]
